# Spectral Properties of Er^3+^/Tm^3+^ Co-Doped ZBLAN Glasses and Fibers

**DOI:** 10.3390/ma10050486

**Published:** 2017-05-03

**Authors:** Xili Liao, Xiaobo Jiang, Qiuhong Yang, Longfei Wang, Danping Chen

**Affiliations:** 1 Department of Materials Science and Engineering, Shanghai University, Shanghai 200444, China; liaobc2010@163.com (X.L.); pubjob_jiang@163.com (X.J.); yangqiuhong@shu.edu.cn (Q.Y.); 2 Key Laboratory of Materials for High Power Laser, Shanghai Institute of Optics and Fine Mechanics, Chinese Academy of Sciences, Shanghai 201800, China; wang_lf@siom.ac.cn

**Keywords:** Er^3+^/Tm^3+^, broadband, ZBLAN, fluoride fiber

## Abstract

A series of Er^3+^/Tm^3+^ co-doped fluoride (ZBLAN) glasses and fibers was prepared and their fluorescence spectra was measured under excitation at 793 nm and 980 nm. Correlation between the self-absorption effect of rare-earth ions and the shift of the emission peak was investigated. With the increasing length of fiber, the emission peaks red-shift when self-absorption occurs at the upper level of emission transition or blue-shift when that occurs at the lower level. As a result of the strong self-absorption effect, Er^3+^/Tm^3+^ co-doped fibers mainly yield 1390–1470, 1850–1980, and 2625–2750 nm emissions when excited at 793 nm, and 1480–1580, 1800–1980, and 2625–2750 nm emissions when excited at 980 nm. Further, a broadband emission in the range of 1410–1580 nm covering the S + C communication band was obtained by the dual-pumping scheme of 793 nm and 980 nm. Results suggest that the dual-pumping scheme would be more effective and important for an Er^3+^/Tm^3+^ co-doped fiber amplifier working in the S + C communication band.

## 1. Introduction

In recent decades, Er^3+^/Tm^3+^ co-doped glasses such as tellurite [1], germanate [2], and bismuthate glasses [3] have been extensively investigated. Er^3+^/Tm^3+^ co-doped glasses have the potential to be drawn into fibers as amplifiers for wavelength division multiplexing (WDM) systems [4,5,6,7]. For multi-channel WDM transmission, it is essential to have a flat-gain broadband spectrum to minimize channel-to-channel crosstalk and gain excursion [8,9,10]. However, the bandwidths of the conventional erbium-doped fiber amplifier (EDFA) for the C-band (1530–1565 nm) and the thulium-doped fiber amplifier (TDFA) for the S-band (1460–1530 nm) are limited [11,12]. A logical approach to achieving broadband gain covering the S + C band is to dope with both Er^3+^ and Tm^3+^ [13,14,15,16]. Additionally, Er^3+^/Tm^3+^ co-doped glass is regarded as one of the approaches to quench the lower level and enhance MIR emission due to the Er^3+^:^4^I_13/2_→Tm^3+^:^3^F_4_ energy transfer process [17,18].

However, because of the long-range interaction between light and materials in fibers, emission characteristics may be very different to those in bulk glasses. So far, only silicate and phosphor glasses have been used as laser glass [19]. Other rare-earth doped glasses are yet to be drawn into fiber. Research on the emission characters of rare-earth ions in fibers would be more important than those in glasses.

In this paper, fluoride glass (ZBLAN [20]) was chosen as the host material because of its low max phonon energy (~500 cm^−1^) to achieve population inversion between the Tm^3+^:^3^H_4_ and ^3^F_4_ levels as well as the Er^3+^:^4^I_11/2_ and ^4^I_13/2_ levels [21]. Consequently, a series of Er^3+^/Tm^3+^-doped ZBLAN glasses with different concentrations and 0.2 mol % Er^3+^–1.2 mol % Tm^3+^ co-doped ZBLAN fibers of different lengths were prepared. Their fluorescence spectra were measured under excitation at 793 nm and 980 nm. Spectral differences between glasses and fibers, as well as the energy transfer mechanisms between Er^3+^ and Tm^3+^, have been discussed. Broadband emission in the range 1410–1580 nm covering the S + C communication band is obtained by the dual-pump scheme of 793 nm and 980 nm.

## 2. Experimental

The investigated ZBLAN glasses had the following molar composition: 53ZrF_4_–20BaF_2_–(3.8 − *x*)LaF_3_–3AlF_3_–20NaF–0.2ErF_3_–*x*TmF_3_ (*x* = 0, 1.2 designated E0.2, E0.2T1.2, respectively). A 0.4 mol % TmF_3_ single-doped glass was also prepared for comparison and designated T0.4. Well-mixed 15 g batches of the initial metal fluoride powders (purity > 99%) were melted in a platinum crucible at 900 °C for 30 min, quenched onto a preheated copper mold and annealed at 230 °C. Finally, the obtained glasses were processed and polished to 15 × 15 × 2 mm^3^ for optical measurements. The fiber preform doped with 0.2 mol % ErF_3_ and 1.2 mol % TmF_3_, was fabricated by quenching molten glass into a polyfluoroalkoxy (PFA) tube in a cold water bath. After annealing at 230 °C, composite of glass rod and PFA tube was drawn together into 300 μm in a fiber drawing tower at 270 °C in the atmosphere of nitrogen.

Refractive index of plain glass was recorded 1.49 at 1552 nm by the prism minimum deviation method. The characteristic temperature of plain glass was measured by a NetzschSTA449/C differential scanning calorimeter (Selb, Germany) at a heating rate of 10 K/min. Absorption spectra of the glasses were measured with a Perkin-Elmer Lambda 900 UV/VIS/NIR spectrophotometer (Llantrisant, UK) in the range 400–2000 nm. The emission spectra were measured with an Edinburgh Instruments FLSP 920 spectrometer (Livingston, UK). Emission spectra of fluoride fibers were measured with a Flight Technology FLA4000 miniature fiber optic spectrometer (Hangzhou, China) in the visible region and a Zolix Instruments Omni-λ 300 spectrometer (Beijing, China) attached with a Scitec Instruments 420 dual phase lock-in amplifier (Trowbridge, UK). Considering the limitations of detectors, the spectra were recorded with an InGaAs detector in the range 1300–1650 nm, a StellarNet RED-Wave NIRx spectrometer (Tampa, FL, USA) in the range 1650–2200 nm and a liquid nitrogen cooling InSb detector in the range of 2580–2800 nm. All measurements were carried out at room temperature.

## 3. Results and Discussion

### 3.1. Thermal Stability

**Figure 1:**
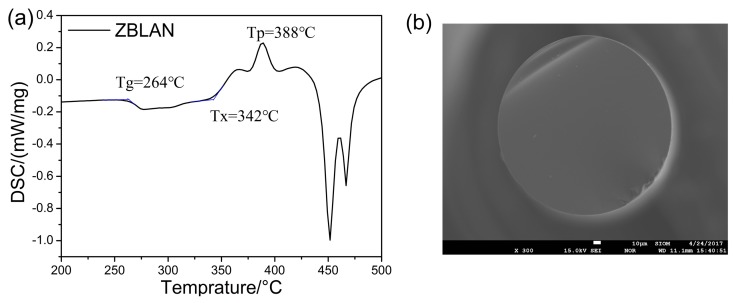
(**a**) Differential scanning calorimeter (DSC) result of ZBLAN glass; (**b**) Cross-sectional view of fiber without PFA coating by EPMA.

Differential scanning calorimeter (DSC) result is shown in Figure 1a. Characteristic temperatures of *T_g_* (temperature of glass transition), *T_x_* (temperature of onset of crystallization), and *T_p_* (temperature of peak of crystallization) are 264, 342, and 388 °C. According to these characteristic temperatures, the fiber preform is drawn at 270 °C in the atmosphere of nitrogen and the cross-sectional view of fiber without PFA coating is obtained by electron probe micro-analyzer (EPMA) as shown in Figure 1b.

### 3.2. Absorption Spectra

**Figure 2:**
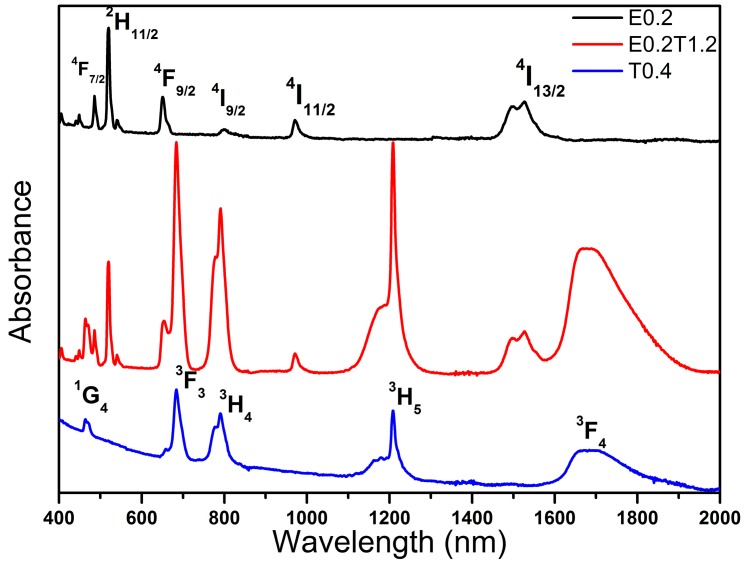
Absorption spectra of Er^3+^/Tm^3+^-doped glasses.

Absorption spectra of Er^3+^-, Tm^3+^-, and Er^3+^/Tm^3+^-doped ZBLAN glasses in the wavelength region 400–2000 nm are presented in Figure 2. Absorption bands corresponding to the transitions from the ground states to specified levels are labeled in the figure. The overlap band centered at ~800 nm is attributed to absorptions from the ground state ^4^I_15/2_ to ^4^I_9/2_ of Er^3+^ and ^3^H_6_ to ^3^H_4_ of Tm^3+^. Other absorption bands are individually related to the Er^3+^ or Tm^3+^. The absorption coefficients are proportional to the concentrations of Er^3+^ and Tm^3+^.

### 3.3. Emission Spectra and Energy Transfer Mechanism

**Figure 3:**
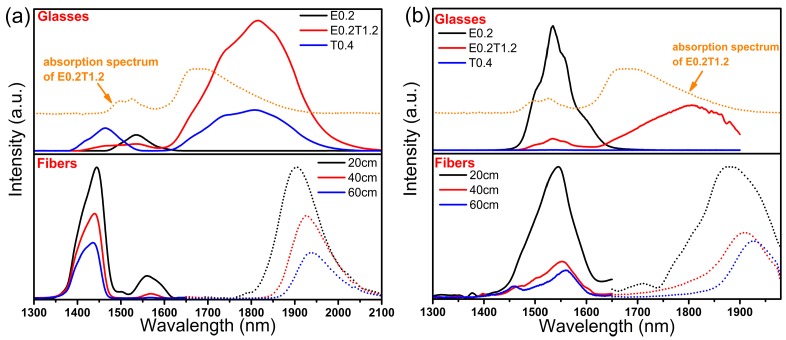
Near-infrared emission spectra of glasses (with different concentrations) and fibers (with different lengths) when excited at (**a**) 793 nm and (**b**) 980 nm.

**Figure 4:**
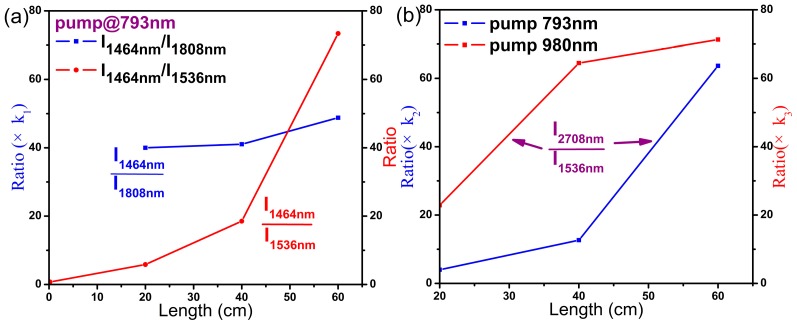
Emission intensity ratios of (**a**) I_1464 nm_/I_1536 nm_ and I_1464 nm_/I_1808 nm_ under 793 nm excitation; and (**b**) I_2708 nm_/I_1536 nm_ when excited at 793 nm and 980 nm.

Figure 3 illustrates the near-infrared (NIR) emission spectra of the three glass samples and the co-doped fibers excited at (a) 793 nm and (b) 980 nm in the range of 1300–2100 nm. It shows that the emission spectra of the co-doped fibers are different from those of the glass samples. In co-doped glass the energy transfer processes Tm^3+^:^3^H_4_ → Er^3+^:^4^I_9/2_ and Er^3+^:^4^I_13/2_ → Tm^3+^:^3^F_4_ attenuate the emissions at 1464 nm and 1536 nm and increase the emission at 1808 nm, as reported previously [22]. In fibers (Figure 3a) excited at 793 nm, the emission intensity of Er^3+^:^4^I_13/2_ → ^4^I_15/2_ decreases rapidly and peak position shifts to longer wavelengths with increasing length of fiber. Emissions of Tm^3+^ are also decreased and accompanied by a blue-shift (for ^3^H_4_ → ^3^F_4_) or a red-shift (for ^3^F_4_ → ^3^H_6_). In fibers excited at 980 nm (Figure 3b), the population of the Tm^3+^:^3^H_4_ level most likely originates from the up-conversion of Er^3+^ and energy transfer between Er^3+^ and Tm^3+^. The emissions are observed to change with increasing length of fiber in a manner similar to that when excited at 793 nm due to the high background loss of fiber (around 5 dB/m) and significant self-absorption effect of rare-earth ions. To determine which factor is more prominent, intensity ratios of I_1464 nm_/I_1536 nm_ and I_1464 nm_/I_1808 nm_ following excitation at 793 nm were computed, as shown in Figure 4a. Because the emission at 1808 nm is recorded on another detector and is normalized individually, the ratio of I_1464 nm_/I_1808 nm_ should be multiplied by an uncertain factor *k*_1_. Both ratios increase with increasing length of fiber, which indicates that self-absorption effects of Er^3+^:^4^I_15/2_ → ^4^I_13/2_ and Tm^3+^:^3^H_6_ → ^3^F_4_ are prominent and cause differences in fluorescence between glasses and fibers. Thus, it would not be proper to infer the emission performance of fibers only from the spectra of glasses.

**Figure 5:**
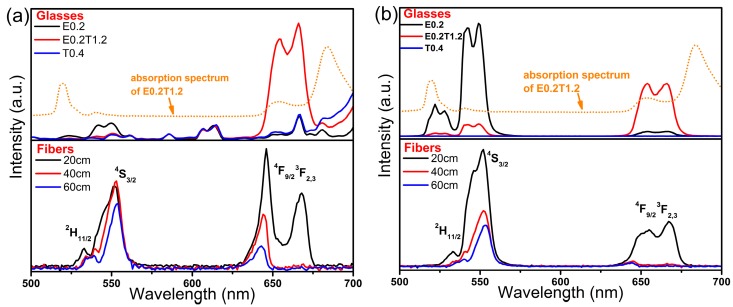
Mid-infrared emission spectra of glasses and fibers excited at (**a**) 793 nm (or 808 nm for glasses) and (**b**) 980 nm.

Mid-infrared (MIR) spectra of glasses and fibers in the range 2580–2800 nm were also obtained as shown in Figure 5 excited at 793 nm ((a), or 808 nm LD for glass samples) and (b) excited at 980 nm. The ascendant long-wavelength tails of the spectra in Figure 5a can be attributed to secondary diffraction of the Tm^3+^:^3^H_4_ → ^3^F_4_ emission. Doping with Tm^3+^ increases the emission intensity at 2708 nm in glasses excited at 808 nm due to the Tm^3+^:^3^H_4_ → Er^3+^:^4^I_9/2_ and Er^3+^:^4^I_13/2_ → Tm^3+^:^3^F_4_ energy transfer processes. Conversely, it decreases the emission intensity when excited at 980 nm. MIR spectra of the co-doped fibers are coincident with the glass samples excited at 793 nm or 980 nm. I_2708 nm_/I_1536 nm_ ratios after excitation at 793 nm and 980 nm were also calculated (Figure 4b). In fact, the values should be multiplied by uncertain factors *k*_2_ and *k*_3_ since they used different detectors. Although the emission intensity at 2708 nm decreases with increasing length of fiber due to the background loss of fiber and absorption by hydroxyl, the ratio of I_2708 nm_/I_1536 nm_ rises, which indicates that the self-absorption effect Er^3+^:^4^I_15/2_ → ^4^I_13/2_ is significant under both excitations. The results of Figure 4 imply that self-absorption from ground state is more prominent than background loss and the emissions with self-absorption will drastically attenuate with increasing fiber length.

**Figure 6:**
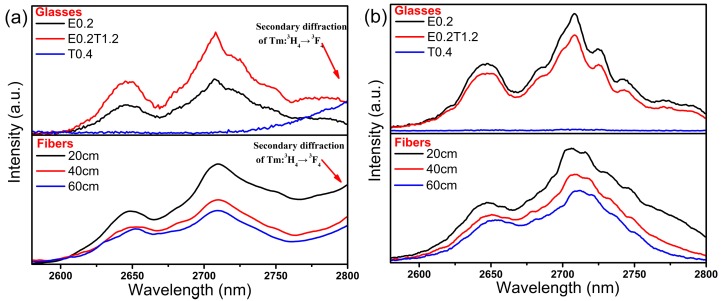
Up-conversion emission spectra of glasses and fibers excited at (**a**) 793 nm (or 808 nm for glasses); and (**b**) 980 nm.

To further understand the energy transfer mechanisms, up-conversion (UC) spectra of all samples were measured with excitation at 793 nm (or 808 nm for glass samples) and 980 nm, as shown in Figure 6. For glasses, excitation at 808 nm generate observable red emissions attributed to Tm^3+^:^3^F_2,3_ → ^3^H_6_ and Er^3+^:^4^F_9/2_ → ^4^I_15/2_ when the concentration of Tm^3+^ is 1.2 mol %. However, exciting at 980 nm can generate the green emission related to Er^3+^:^2^H_11/2_, ^4^S_3/2_ → ^4^I_15/2_ and the red emission related to the Er^3+^:^4^F_9/2_ → ^4^I_15/2_ and Tm^3+^:^3^F_2, 3_ → ^3^H_6_ transitions. For co-doped fibers excited at 793 nm, the green emission at 550 nm and the red emission at 660 nm can be obtained due to the higher optical power density of the LD pump light. With 980 nm excitation, the green emission is much stronger than the red emission, contrary to that found in glass samples. Red emissions at both excitation wavelengths are significantly decreased and shift to shorter wavelengths with increasing length of fiber due to the self-absorption effect of Tm^3+^:^3^H_6_ → ^3^F_2, 3_.

**Figure 7:**
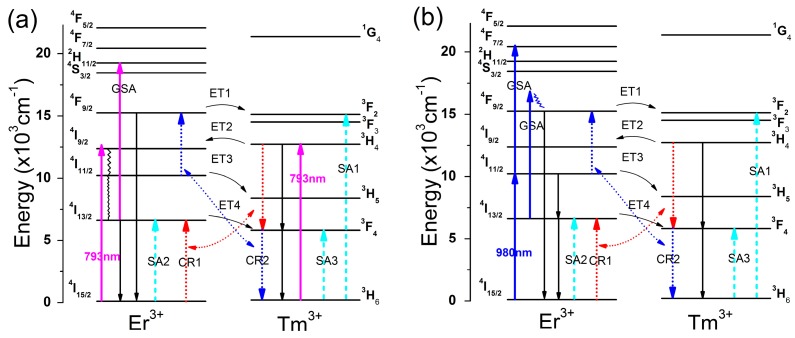
Energy level diagrams of Er^3+^ and Tm^3+^ and energy transfer mechanisms for excitation at (**a**) 793 nm and (**b**) 980 nm.

To demonstrate the energy transfer mechanisms between Er^3+^ and Tm^3+^, the energy level diagrams of Er^3+^ and Tm^3+^ are given in Figure 7, for excitation at (a) 793 nm and (b) 980 nm. With excitation at 793 nm, ground state absorption (GSA) occurs for Er^3+^:^4^I_15/2_ → ^4^I_9/2_ and Tm^3+^:^3^H_6_ → ^3^H_4_. The ^4^I_9/2_ level relaxes to the ^4^I_11/2_ level by multi-phonon relaxation and further relaxes to the ^4^I_13/2_ level by radiative or non-radiative relaxation. Part of the population of the ^4^I_13/2_ level de-excites to the ^4^I_15/2_ level, causing a 1536 nm emission. Another part may be excited to the ^2^H_11/2_ level by excited state absorption (ESA), resulting in 525, 550, and 660 nm emissions due to de-excitations of the ^2^H_11/2_, ^4^S_3/2_, and ^4^F_9/2_ levels, respectively. For Tm^3+^, the ^3^H_4_ level relaxes to the ^3^F_4_ level, resulting in 1464 nm emission and further relaxes to the ground state ^3^H_6_ level, resulting in 1808 nm emission. There are several possible energy transfer processes between Er^3+^ and Tm^3+^ following 793 nm excitation:

ET1: Er^3+^:^4^F_9/2_ → ^4^I_15/2_, Tm^3+^:^3^H_6_ → ^2^F_2, 3_, 222 cm^−1^ ET2: Er^3+^:^4^I_15/2_ → ^4^F_9/2_, Tm^3+^:^3^H_4_ → ^3^H_6_, 249 cm^−1^ ET3: Er^3+^:^4^I_11/2_ → ^4^I_15/2_, Tm^3+^:^3^H_6_ → ^3^H_5_, 1922 cm^−1^ ET4: Er^3+^:^4^I_13/2_ → ^4^I_15/2_, Tm^3+^:^3^H_6_ → ^3^H_4_, 892 cm^−1^ CR1: Er^3+^:^4^I_15/2_ → ^4^I_13/2_, Tm^3+^:^3^H_4_ → ^3^F_4_, 408 cm^−1^ CR2: Er^3+^:^4^I_11/2_ → ^4^F_9/2_, Tm^3+^:^3^F_4_ → ^3^H_6_, 583 cm^−^^1^

The prominent self-absorption processes follow:

SA1: Tm^3+^:^3^H_6_ → ^3^F_2, 3_ SA2: Er^3+^: ^4^I_15/2_ → ^4^I_13/2_ SA3: Tm^3+^:^3^H_6_ → ^3^F_4_

The ET1 process, which is a quasi-resonant energy transfer process with an energy mismatch of 222 cm^−1^ populates the Tm^3+^:^3^F_2, 3_ levels resulting in enhancement of the 660 nm emission with increasing concentration of Tm^3+^. The ET2 process is a quasi-resonant energy transfer process with an energy mismatch of 249 cm^−1^, which depopulates the Tm^3+^:^3^H_4_ level and results in a reduction of the emission intensity at 1464 nm. ET3 and ET4 are non-resonant processes that depopulate the Er^3+^:^4^I_11/2_ and Er^3+^:^4^I_13/2_ levels resulting in reduced emission at 1536 nm. ET3 and ET4 populate the Tm^3+^:^3^H_5_ (which decays non-radiatively to the ^3^F_4_ level) and Tm^3+^:^3^F_4_ levels and consequently enhance the emission at 1808 nm. By cross-relaxation process CR1, the Tm^3+^:^3^H_4_ level relaxes to ^3^F_4_ and transfers energy to the Er^3+^:^4^I_13/2_ level. CR1 decreases the emission intensity at 1464 nm and enhances the emission at 1536 nm. By the CR2 process, the Tm^3+^:^3^F_4_ level relaxes to ^3^H_6_ and transfers energy to Er^3+^ exciting the ^4^I_11/2_ level to the ^4^F_9/2_ level, resulting in enhancement of the red emission. The CR1 and CR2 processes possibly involve participation of one or two phonons at room temperature due to small energy mismatches of 408 cm^−1^ and 583 cm^−1^, respectively. The self-absorption processes SA1, SA2, and SA3 drastically decrease the emission intensities of 660, 1536, and 1808 nm respectively, and shift the peak positions.

With 980 nm excitation, the Er^3+^:^4^I_11/2_ level is populated by GSA and then relaxes to ^4^I_13/2_ with emission at 2708 nm. ESA produces transitions of Er^3+^:^4^I_11/2_ → ^4^F_7/2_ and ^4^I_13/2_ → ^4^F_9/2_ resulting in up-conversion emissions. Energy transfers from Er^3+^ to Tm^3+^ would be more active due to the stronger absorption by Er^3+^ at 980 nm than at 793 nm.

### 3.4. Dual-Pump for Broadband

**Figure 8:**
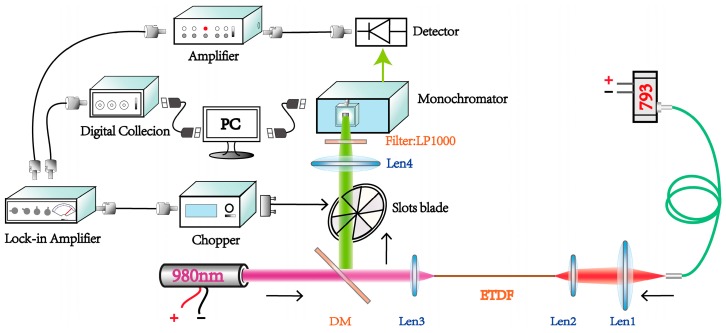
Schematic diagram of dual-pump and backward measurement system.

**Figure 9:**
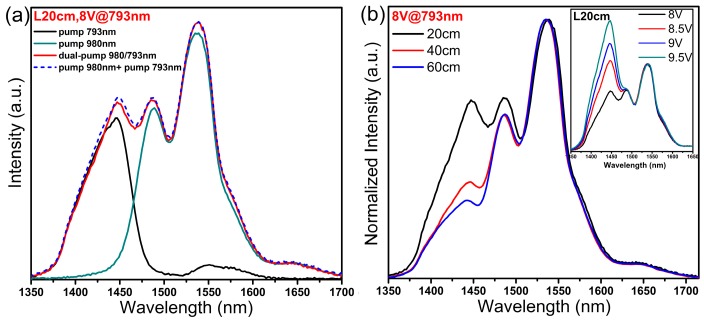
NIR emission (**a**) spectrum of a 20 cm long fiber under 793 nm or 980 nm single-pump, and (**b**) spectra of fibers with varied length under the dual-pump at 793 nm and 980 nm. The driving voltage of 793 LD was fixed to 8 V. The inner picture shows the dual-pump spectrum of 20 cm long fiber.

As illustrated in Figure 3, a single-pump at 793 nm or 980 nm does not generate broadband emission that covers the S + C communication band due to the significant self-absorption effect of Er^3+^. Hence, a dual-pumping scheme with excitation at both 793 nm and 980 nm was adopted and the experimental schematic diagram is shown in Figure 8. Fixed 980 nm pump light was launched into the trailing end of the Er^3+^/Tm^3+^ co-doped fiber to compensate for the loss of emission at 1530 nm. Meanwhile, a dichroic mirror was used to reflect the light produced from 1400–1650 nm (reflection > 99%). The NIR spectra obtained are shown in Figure 9. Figure 9a demonstrates that the broadband obtained by dual-pumping is the combination of emissions by single-pumping of 793 nm and 980 nm. While the pump power is fixed, the emission intensity of 1447 nm rises with increasing length of fiber, as shown in Figure 9b. Additionally, for a selected 20 cm long fiber, emission at 1447 nm is enhanced by increasing the driving voltage of the 793 nm LD, as shown in the inner figure. As a result, compensation of the 1530 nm emission by launching into the trailing end of the fiber with 980 nm LD is effective in producing a broadband emission. This also shows a convenient way to modify the shape of the fluorescence spectrum by changing the pump power and length of fiber.

## 4. Conclusions

A series of Er^3+^/Tm^3+^ co-doped fluoride (ZBLAN) glasses and fibers was prepared and their fluorescence spectra were measured under excitation at 793 nm and 980 nm. Correlation between the self-absorption effect of rare-earth ions and emission peak shift was investigated. With increasing length of fiber, the emission peaks red-shift when self-absorption occurs at the upper level of emission transition or blue-shift when it occurs at the lower level. This suggests that it would not be proper to infer the emission performance of fibers only from the spectra of glasses due to the strong self-absorption effect of rare-earth ions. Er^3+^/Tm^3+^ co-doped fibers—due to the significant self-absorption effect of rare-earth ions—mainly yield 1390–1470, 1850–1980, and 2625–2750 nm emissions with excitation at 793 nm, and 1480–1580, 1800–1980, and 2625–2750 nm emissions with excitation at 980 nm. Compensating for the loss of Er^3+^:^4^I_13/2_ → ^4^I_15/2_ due to the self-absorption effect, broadband emission in the range 1410–1580 nm was obtained by dual-pumping at 793 nm and 980 nm. Therefore, a dual-pumping scheme would be more effective and important for an Er^3+^/Tm^3+^ co-doped fiber amplifier working in the S + C communication band.
